# Metabolic engineering of *Saccharomyces cerevisiae* for hydroxytyrosol overproduction directly from glucose

**DOI:** 10.1111/1751-7915.13957

**Published:** 2021-10-24

**Authors:** Ricardo Bisquert, Andrés Planells‐Cárcel, Elena Valera‐García, José Manuel Guillamón, Sara Muñiz‐Calvo

**Affiliations:** ^1^ Departamento de Biotecnología de Alimentos Instituto de Agroquímica y Tecnología de Alimentos IATA‐CSIC Agustín Escardino 7 Paterna Valencia 46980 Spain

## Abstract

Hydroxytyrosol (HT) is one of the most powerful dietary antioxidants with numerous applications in different areas, including cosmetics, nutraceuticals and food. In the present work, heterologous hydroxylase complex HpaBC from *Escherichia coli* was integrated into the *Saccharomyces cerevisiae* genome in multiple copies. HT productivity was increased by redirecting the metabolic flux towards tyrosol synthesis to avoid exogenous tyrosol or tyrosine supplementation. After evaluating the potential of our selected strain as an HT producer from glucose, we adjusted the medium composition for HT production. The combination of the selected modifications in our engineered strain, combined with culture conditions optimization, resulted in a titre of approximately 375 mg l^−1^ of HT obtained from shake‐flask fermentation using a minimal synthetic‐defined medium with 160 g l^−1^ glucose as the sole carbon source. To the best of our knowledge, this is the highest HT concentration produced by an engineered *S. cerevisiae* strain.

## Graphical abstract

In the present work, our aim has been to engineer an HT‐overproducing yeast strain directly from a simple carbon source like glucose in *S. cerevisiae*.

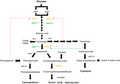

## Introduction

Both virgin olive oil and wine are rich in polyphenols, including tyrosol, oleuropein, hydroxytyrosol (HT), elenolic acid and resveratrol (Carrasco‐Pancorbo *et al*., 2005; Fernández‐Mar *et al*., 2012). Of these, HT is a natural antioxidant considered to be one of the main ingredients that promotes health and a bioactive component in Mediterranean diet characterized by regular olive oil intake (Daniele *et al*., 2017). Several studies have demonstrated extensive biological HT properties with both *in‐vitro* and *in‐vivo* models (D’Angelo *et al*., 2020). The ability to cross the brain barrier and its high bioavailability and degree of absorption, together with the health claim approved by EFSA (Turck *et al*., 2017), highlight the importance of this polyphenol for food, feed, supplement and pharmaceutical industries (Britton and Davis, 2019). However, the price of commercially available pure HT forms can be high, which makes its use in the food industry economically unviable (Achmon and Fishman, 2015).

The main ways to obtain HT are plant extraction or chemical synthesis. As olive tree derivatives are the most accessible source, the majority of HT products come from the extraction of olives or olive oil waste streams, of which the latter is a favourable source because it originates from a by‐product. However, HT extraction from any of these sources is a lengthy process that yields low recovery rates, which can vary seasonally from batch to batch. Chemical synthesis methods usually involve non environmentally friendly solvents and expensive starting substrates, which sometimes make it unsuitable for large‐scale industrial production (Zhang *et al*., 2012; Achmon and Fishman, 2015; Britton *et al*., 2019). Therefore, biotechnological HT production can potentially be the dominant production process for the future. Whole‐cell catalysts for HT biosynthesis have been used with different bacterial microorganisms (Liebgott *et al*., 2009; Bouallagui and Sayadi, 2018; Li *et al*., 2018; Hassing *et al*., 2019; Yao *et al*., 2020). Specially remarkable is the use of *Escherichia coli*, in which the co‐expression of yeast *ARO10* and *ADH6* genes, and the overexpression of HpaBC, produced important amounts of HT (Chung and Kim, 2017; Li *et al*., 2018). Recently, through structure‐guided modelling and directed evolution, the HpaBC complex was used as tyrosine hydroxylase instead of tyrosol hydroxylase, leading to a 95% conversion rate of tyrosine to L‐DOPA. This strategy yields a remarkably high HT production using tyrosine as a first substrate via L‐DOPA decarboxylase, tyramine oxidase (TYO) and alcohol dehydrogenase using an *in vivo* evolved TYO (Yao *et al*., 2020). Baker’s yeast, *Saccharomyces cerevisiae*, is also a promising cell factory for producing recombinant HT thanks to a number of advantages, such as robust growth on simple media, feasibility in genetic manipulations and it is ‘generally regarded as safe’ (GRAS) (Nielsen *et al*., 2013; Guo *et al*., 2019).

Our previous work involved the heterologous overexpression of genes *hpaB* and *hpaC* from *E. coli* in *S. cerevisiae* by using episomal plasmids (Muñiz‐Calvo *et al*., 2020). Through this overexpression, we achieved titres ranging from 1.15 to 4.6 mg l^−1^ in a minimal medium in which either 1 mM tyrosine or 1 mM tyrosol was, respectively, added, with tyrosol being the preferred starting material. Nevertheless, tyrosol and tyrosine cost approximately 8720 and 2000 € kg^−1^ provided by the same supplier (Merck, Darmstadt, Germany), which is around 281‐ and 64‐fold higher than that of glucose (31 € kg^−1^), making this monosaccharide a more appealing source for producing HT. Thus the present work aimed to engineer an HT‐overproducing yeast strain *S. cerevisiae* directly from a simple carbon source like glucose. We first constructed a plasmid‐free yeast strain harbouring the HpaBC complex integrated into multiple copies, and used metabolic engineering to direct the carbon flow to HT production. We also achieved higher product titres by optimizing the growth conditions during shake‐flask fermentations.

## Results and discussion

### Optimization of the hydroxylation of tyrosol into HT

We have previously demonstrated that the overexpression of genes *hpaB* and *hpaC* from *E. coli* in yeast is a promising tool to overproduce HT. However, the maximum amounts obtained by our former strain (4.6 ± 0.9 mg l^−1^) were in a minimal medium that required tyrosol supplementation in the medium (Muñiz‐Calvo *et al*., 2020). After this proof of concept, our interest focussed on improving HT production from glucose as a substrate.

We first decided to integrate both genes into the yeast genome before any further modification to achieve greater stability in the expression of those genes compared to the overexpression with episomal plasmids. We constructed integrative cassette HpaBC, which contained *hpaC* with the *TEF1* promoter and *hpaB* with the *PGK1* promoter, and we used multi‐integrative plasmid pCfB2988 from the EasyCloneMulti series (Maury *et al*., 2016). This vector allows the simultaneous integration into the yeast genome of both genes in multiple copies because it presents sequences with a homology to Ty1 elements.

After integrating the pCf2988 containing HpaBC in BY4743, we evaluated the HT production of 24 different transformants in SC with 1 mM of tyrosol (Fig. S1). Interestingly, although all the colonies showed HT production, a clear difference in production was observed in the different clones transformed with the integrative HpaBC cassette. This can be explained by the fact that each transformant could have integrated a different copy number of the HpaBC complex. Furthermore, most of the tested colonies produced much more HT (over 10 mg l^−1^) than the amounts previously obtained by the overexpression of both genes in plasmids (Fig. S1). These results make sense because it has been previously shown that a smaller fraction of the cell population exhibits a high expression of the gene of interest when overexpressed with episomal plasmids compared to integration (Maury *et al*., 2016) due to segregational instability (Jensen *et al*., 2014). Thus we selected the most productive transformant (henceforth named HpaBC) to explore additional modifications to increase HT synthesis from glucose.

### Metabolic engineering of the shikimate and Ehrlich pathways to increase tyrosol and HT production

**Fig. 1:**
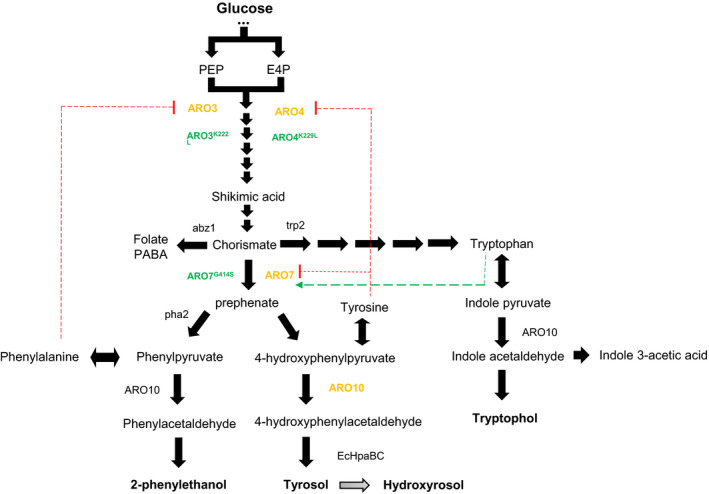
Overview of the aromatic amino acid metabolism in *Saccharomyces cerevisiae*. Hydroxytyrosol was heterologously produced from glucose through the shikimate and Ehrlich pathways, together with the *Escherichia coli* hydroxylase complex (EcHpaBC). The overexpression of the native or engineered enzymes is indicated in yellow and green respectively. The dotted red lines indicate allosteric inhibition by the phenylalanine of *ARO3* and by the tyrosine of *ARO4* and *ARO7*, whereas the green dotted line denotes *ARO7* activation by tryptophan. Knock‐out tested are indicated in lower case letters, including para‐aminobenzoate (PABA) synthase (*ABZ1*), anthranilate synthase (*TRP2*) and prephenate dehydratase (*PHA2*). The measured metabolites are shown in bold. This figure is adapted from Cordente *et al*. (2019).

In *S. cerevisiae*, tyrosol can be obtained from tyrosine through the Ehrlich pathway (Ehrlich, 1907; Sentheshanmuganathan and Elsden, 1958) (Fig. 1). Indeed the yeast genes involved in these reactions have been heterologously expressed in *E. coli* to overproduce tyrosol and HT (Xue *et al*., 2017; Li *et al*., 2018). However, a recent study has shown that α‐keto acid precursors, required for the *de novo* synthesis of aromatic higher alcohols, come mainly from the catabolism of sugars through the shikimate pathway, with a limited contribution from the anabolism of consumed amino acids (Crépin *et al*., 2017).

Given the potential of the HpaBC strain to hydroxylate tyrosol, we decided to increase the flux towards this endogenous precursor for HT synthesis and we followed different approaches to do so. The first approach was to evaluate the effect of the gene‐knockouts of the competitive pathways for tyrosol synthesis through the chorismate metabolism. The second approach was the overexpression of individual ARO genes, and their feedback‐resistant derivatives forms. Finally, we also evaluated the effect of the combined overexpression of ARO genes at tyrosol levels.

### Effect of the gene‐knockouts of competitive pathways for tyrosol synthesis

The competitive genes that were studied for tyrosol synthesis in the superpathway of chorismate metabolism were *TRP2*, *PHA2* and *ABZ1*. *TRP2* encodes an anthranilate synthase, which catalyses the initial tryptophan biosynthesis step (Fantes and Roberts, 1976), *ABZ1* encodes a *para*‐aminobenzoate synthase involved in the synthesis of *p*‐aminobenzoic acid from chorismate, but has also been related to 2‐phenylethanol production (Edman and Goldstein, 1993; Steyer *et al*., 2012), and *PHA2* encodes prephenate dehydratase, which consumes prephenate in the phenylalanine biosynthesis pathway (Maftahi *et al*., 1995). All these genes are represented in Fig. 1. Unexpectedly the deletion of these genes, which encode the enzymes competing for a common substrate, did not increase the chorismate flow towards the synthesis of both tyrosine and tyrosol. No significant improvement in tyrosol production was observed in the Δ*trp2* strain, and even a decrease was detected for mutant strains Δ*abz1* and Δ*pha2* (Fig. S2) compared with the wild‐type strain BY4743. Given that the single deletion of those genes was not an optimal strategy to increase tyrosol production in *S. cerevisiae*, we decided to follow a different strategy, which is described in the next section.

### Effect of individual ARO genes overexpression on tyrosol and HT production

**Fig. 2:**
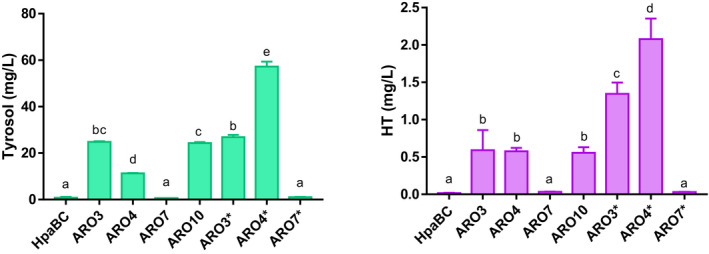
Effect of the single overexpression of several genes involved in amino acid metabolism on tyrosol and hydroxytyrosol production. HpaBC strain was transformed with the empty p423GPD plasmid (control) or with p423GPD containing one of the following genes: *ARO3, ARO4*, *ARO7*, *ARO10*, ARO3*, ARO4* and ARO7*. Each strain harbouring one plasmid was cultured for 72 h at 30 °C in SD +leu. Tyrosol and hydroxytyrosol were determined from the supernatant extracted with methanol and analysed by UHPLC‐MS/MS. The values under the same letter are not significantly different according to the Tukey HSD test.

Tyrosol is naturally produced from the catabolism of amino acids via the Ehrlich pathway in *S. cerevisiae,* and phenylpyruvate decarboxylase *ARO10* catalyses the entrance reaction in this pathway by the conversion of 4‐hydroxyphenylpyruvate (4HPP) into 4‐hydroxyphenyl acetaldehyde (4HPAA) (Fig. 1). *ARO10* overexpression in *E. coli* leads to high *de novo* tyrosol production in this microorganism (Xue *et al*., 2017; Xu *et al*., 2020). Therefore, we decided to overexpress *ARO10* in BY4743 and evaluate the effect on tyrosol production. As shown in Fig. 2 and Table S4, *ARO10* overexpression increased 45.5‐fold the tyrosol levels in SD medium. This increase in tyrosol levels resulted in 40‐fold higher HT production for this strain compared to the HpaBC strain.

After checking the potential of *ARO10* to increase the synthesis of both tyrosol and HT, we aimed to examine other modifications upstream of the Erhlich pathway that could increase the endogenous pool of HT precursors. The first amino acid biosynthesis step is the condensation of phosphoenolpyruvate (PEP) and erythrose 4‐phosphate (E4P) to form 3‐deoxy‐D‐arabino‐heptulosonate‐7‐phosphate (DAHP) via the shikimate pathway. This step is performed by one of the two DAHP synthase isozymes *ARO3* and *ARO4* (Teshiba *et al*., 1986). The chorismate mutase, encoded by *ARO7,* catalyses the conversion of chorismate into prephenate (Ball *et al*., 1986), the last precursor common to both phenylalanine and tyrosine (Fig. 1). Our results showed that the individual overexpression of either *ARO3* or *ARO4* was successful for raising tyrosol levels, and achieved an improvement of about 50‐ and 21‐time fold respectively. The single *ARO7* overexpression did not improve tyrosol levels (Fig. 2, Table S4).

Regarding HT, the overexpression of *ARO3* or *ARO4* also increased the production of this molecule, similarly to the concentration achieved by *ARO10* overexpression (Fig. 2, Table S4). Interestingly, *ARO7* overexpression led to a 2‐fold higher HT levels despite tyrosol levels not increasing (Fig. 2, Table S4).

### Effect of individual overexpression of deregulated ARO3*, ARO4* and ARO7* on tyrosol and HT production

The above ARO wild‐type overexpression improved both tyrosol and HT production. However, it is well known that *ARO3* is allosterically inhibited by phenylalanine, whereas *ARO4* by tyrosine and, likewise *ARO7*, have been identified as being subjected to allosteric regulation in a subsequent reaction, and inhibited by tyrosine and stimulated by tryptophan (Lingens and Goeberl, 1967). Therefore, we decided to overexpress the variants with the abolished feedback inhibition by those products of the shikimate pathway. Previous works state that modified *ARO3^K222L^* (ARO3*), *ARO4^K229L^* (ARO4*) and *ARO7^G141S^* (ARO7*) result in feedback inhibition‐insensitive enzymes (Schmidheini *et al*., 1989; Fukuda *et al*., 1991; Luttik *et al*., 2008). Likewise, different engineered strains include the expressions of these variants to improve the titres of the intermediates of the tyrosine and phenylalanine pathways (Curran *et al*., 2013; Trenchard *et al*., 2015; Brückner *et al*., 2018; Reifenrath and Boles, 2018; Hassing *et al*., 2019). So we examined if the single overexpression of ARO3*, ARO4* and ARO7* had any effect on increasing the pool of the HT precursor, tyrosol, and also on HT production. As shown in Fig. 2, the single overexpression of ARO3* and ARO4* resulted in higher tyrosol levels, and tyrosine feedback‐resistant ARO4* overexpression had the strongest impact (~110‐fold) (Fig. 2, Table S4). The largest amount of tyrosol was also the highest titre of the HT concentration (2.08 ± 0.27 mg l^−1^) achieved by ARO4* overexpression. This concentration represented an increase in HT of 150‐fold (Fig. 2, Table S4). As already happened with the wild‐type allele, ARO7* overexpression produced much less amount of tyrosol and HT than the other ARO genes (Table S4).

### Effect of the combinatorial overexpression of the wild‐type or deregulated versions of ARO genes on tyrosol and HT production

The single overexpression of all the genes resulted in higher tyrosol levels from SD medium, and the *ARO3*, *ARO4*, *ARO10*, ARO3* and ARO4* overexpressions that produced the most (~20 to ~110‐fold increase). To determine whether a possible additive effect on HT production could result from combining the overexpression of some previous genes, up to four ARO genes were overexpressed in the BY4741 Δ*trp1* strain background. The reason for switching from the diploid (BY4743) to the haploid version (BY4741) was that a larger number of auxotrophies is required to overexpress up to four ARO genes in the same strain. Haploid BY4741 harbours four auxotrophic markers (∆*ura3*, ∆*leu2*, ∆*his3*, ∆*met15*), and we additionally deleted the *TRP1* gene to generate a new auxotrophy (Δ*trp1*). Logically, we also constructed strains HpaBC and ARO4* in the BY4741 genetic background, which were used as control strains.

**Fig. 3:**
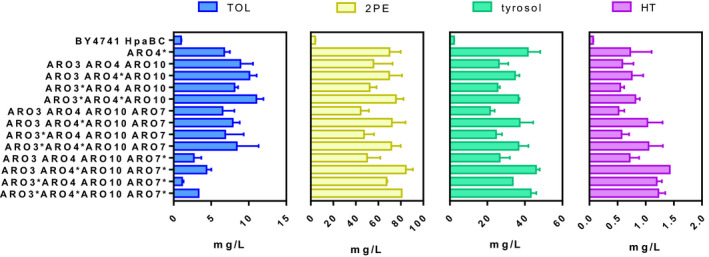
Effect of the combinatorial overexpression of the several genes involved in aromatic amino acid metabolism on aromatic higher alcohols production. BY4741 HpaBC was transformed with different plasmids, each containing an *Saccharomyces cerevisiae* gene for its overexpression (*ARO3, ARO4*, *ARO7*, *ARO10* and feedback‐resistant derivatives ARO3*, ARO4* and ARO7*). The different strains were cultured for 72 h at 30 °C in SD. The tryptophol (TOL), 2‐phenylethanol (2‐PE) and tyrosol concentrations were determined from the supernatant extracted with methanol, and analysed by HPLC‐PDA. The statistical analysis for groups of strains in each detected compound are referred to in Table S5.

In this experiment, together with tyrosol, we also measured the other aromatic higher alcohols (tryptophol and 2‐phenylethanol) to better understand the carbon flux redirection. Figure 3 shows the production of the three aromatic higher alcohols generated by the different engineered strains. Remarkably, the simultaneous overexpression of different ARO genes led aromatic higher alcohols titres to vastly increase compared to control strain HpaBC. Of the three different aromatic higher alcohols, 2‐phenylethanol was produced the most, followed by tyrosol and finally by tryptophol (Fig. 3, Table S5). We detected up to 90 mg l^−1^ of 2‐phenylethanol in one clone of the strain that harboured the combinatorial *ARO3*, ARO4*, ARO10 and ARO7* overexpression. Similar concentrations have been reported by Shen *et al*. (2016) in a yeast strain that overexpressed *ARO10* and *ADH1* combined with the knockout of the *ARO8* gene in the BY4741 background.

The tyrosol level ranged from 20 to 45 mg l^−1^ for the different engineered strains (Fig. 3). Interestingly, the strain that led to the highest 2‐phenylethanol levels (*ARO3*, ARO4*, ARO10 and ARO7*) was the same as that which generated the highest tyrosol concentration, which agrees with the good correlation observed between tyrosol and 2‐phenylethanol production (*r* = 0.9824; Fig. S3). As mentioned above, tyrosol levels drastically dropped with the single overexpression of the *ARO7* wild‐type allele or feedback‐insensitive form ARO7* (Fig. 2). Nonetheless, when this allele, especially ARO7*, was co‐overexpressed with other modifications, including *ARO3*/ARO3*, ARO4* and *ARO10*, tyrosol production increased by about 20‐fold compared to the control strain (Fig. 3). Previous works have already observed a similar phenotype regarding *ARO7* overexpression in glucose‐limited chemostat cultures (Luttik *et al*., 2008).

Finally, tryptophol levels fell within ranges from 1 to 11 mg l^−1^, and the strain that co‐overexpressed ARO3*, ARO4* and *ARO10* was the best producer. Curiously when the modified strains co‐overexpressed ARO7* together with other modifications, tryptophol levels also considerably lowered (Fig. 3). For instance, ARO7* overexpression in the highest tryptophol‐producing strains (ARO3*, ARO4* and *ARO10*) actually lowered tryptophol levels from 11.05 ± 0.95 to 3.33 ± 0.27 mg l^−1^ (Fig. 3). This result can be explained because the tryptophol pathway competes with the pathways of tyrosol and 2‐phenylethanol at the chorismate node. Therefore when *ARO7* is overexpressed, chorismate is used preferentially to produce prephenate and its derivatives 2‐phenylethanol and tyrosol, which results in less chorismate availability for the synthesis of the precursor of tryptophol, namely anthranilate. Insensitive‐feedback allele ARO7* overexpression further enhanced this metabolic flux towards tyrosol and 2‐phenylethanol synthesis. The depletion of chorismate due to *ARO7* and tryptophan‐insensitive *ARO7* allele overexpression has been previously described to lead to tryptophan auxotrophy (Krappmann and Lipscomb, 2000; Luttik *et al*., 2008). However, the observed growth defect was counteracted by transcriptional *TRP2* induction (Krappmann *et al*., 2000). According to our data, we observed no significant difference in the maximum OD_600_ obtained when overexpressing either *ARO7* or ARO7* (data are not shown), but employing *TRP1* as an auxotrophic marker in episomal plasmid should be taken into account because it represents an overexpression of this gene, which could counteract tryptophan limitation by increasing the chorismate pool for anthranilate synthesis.

Regarding HT levels, the different overexpression combinations had a positive impact compared to the control strain HpaBC, and the strain overexpressing *ARO3*, ARO4*, *ARO10* and ARO7* was the highest producer with 1.5 mg l^−1^ of HT, which is practically twice the amount produced by the strain that only overexpressed ARO4* in the haploid version (Fig. 3 and Table S5). However, when we compared the single overexpression of ARO4* in the haploid and diploid background, BY4743 ARO4* synthesized more than 2‐fold HT (2.08 mg l^−1^) than BY4741 ARO4* (0.73 mg l^−1^). Moreover, the single overexpression of ARO4* in BY4743 resulted in higher HT titres than the better combination of the ARO genes in BY4741. As previously reported by Suástegui *et al*. (2016), the production capacity of aromatic compounds can display a wide range of variability in a strain‐dependent manner, even when the same exact genetic modifications are conducted in different strains. These different capacities are closely related to each strain’s ability to adjust copy numbers of episomal vectors, but are also related to differences in pathway balancing to channel carbon metabolism in aromatic amino acid pathways. In any case, in the near future, we will attempt to introduce the best combination of ARO genes for HT production in a diploid background.

### Effect of sugar concentration and fermentation stage on the HT production

**Fig. 4:**
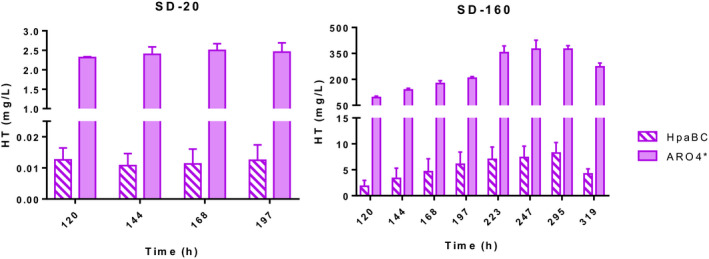
Effect of glucose concentration on hydroxytyrosol production. Strains HpaBC and ARO4* were cultured at 30 °C in shake flasks filled with SD containing 20 and 160 g l^−1^ of glucose (SD‐20 and SD‐160 respectively). Samples were collected at different time points and hydroxytyrosol (HT) concentration was determined from the supernatant extracted with methanol and analysed by HPLC‐PDA. The comparisons between strains HpaBC and ARO4* were significantly different in all time measurements according to Student’s *t*‐test (*P* value ≤ 0.05).

In order to establish suitable growth conditions for HT synthesis, we tested different glucose concentrations to increase HT production. For this purpose, we cultured engineered strains BY4743 HpaBC (control) and BY4743 ARO4* in SD at six glucose concentrations (20, 80, 160, 200, 250 y 300 g l^−1^). HT production was steadily increasing from 20 up to 160 g l^−1^ glucose concentration. Further glucose availability in the growth medium did not result in a significant HT concentration (data not shown). Figure 4 depicts the remarkable glucose concentration effect with HT titres of 375 mg l^−1^ in SD‐160, which is a more than 100‐fold increase compared to the same strain in the medium with 20 g l^−1^ of glucose. As we previously observed a significant rise in the HT production after the 5th day of fermentation, we only show the sampling within a range of times from 120 h to 295 h (Fig. 4). Finally, the fermentation volume was scaled from 1.5 ml (all the above‐explained results were obtained in this volume) to 50 ml. In the conditions explained, the maximum production was obtained from a sampling time of 223 h with no significant increases in further samplings. However, this HT accumulation process in growth medium only happened in SD‐160, and not in SD‐20, which had the same value for all the sampling times (sampling was not continued after 197 h). This result evidenced the dependence of fermentable glucose in the medium on HT synthesis and its precursor tyrosol, but not of its consumption, because less than half of the available sugar was consumed at the end of the fermentation process (75 g l^−1^ of glucose was consumed). Another conclusion reached with this result is that the HT produced and secreted to culture medium remained stable and was either not subsequently metabolized or metabolized at the same rate it was produced, reaching an equilibrium state. Finally, despite the fact that higher OD_600_ values were reached in SD‐160 than in SD‐20 (Fig. S4), this greater growth did not explain the significant differences noted in HT production, but mainly accounted for the enhanced metabolic flux in the pathway of the aromatic amino acids. The molasses is the usual growth medium for the production of biofuels and other bioprocess‐based commodities. In spite of the fact that the composition and final quality of molasses vary a great deal amongst batches, most of them are very rich in sugar content and adequate for optimizing HT production.

Likewise, aromatic higher alcohols accordingly showed a marked increase in SD‐160. The titres of tyrosol, 2‐phenylthanol and tryptophol increased by 2.4, 5.6 and 5.4 for strain ARO4* at 197 h compared to SD‐20 (Fig. S5). The maximum concentration of these aromatic higher alcohols was also reached with the 223‐h sampling. Nonetheless, we wish to point out that, for the first time, we achieved more HT production than its precursor tyrosol for strain ARO4* in a 50 ml culture of SD‐160 after approximately 10 fermentation days.

### Breakthroughs in HT production by different constructed strains

**Fig. 5:**
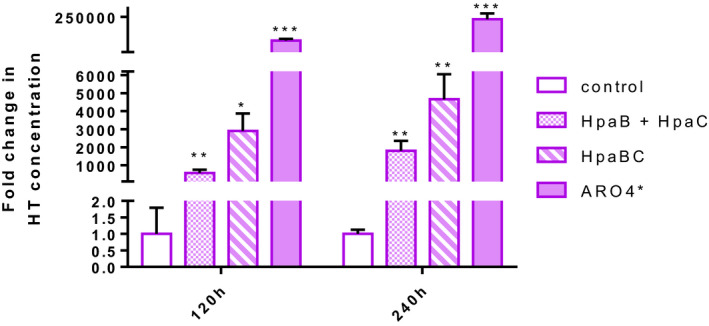
Effect of various genomic modifications on hydroxytyrosol production. The BY4743 control strain (transformed with empty vectors), HpaB + HpaC, HpaBC and ARO4* were cultured in SD medium with 160 g l^−1^ of glucose. Hydroxytyrosol (HT) was measured from the supernatant extracted with methanol at 120 and 240 h, and analysed by HPLC‐PDA. The measured concentrations were normalized to the HT produced by the control strain. Significance levels of Student’s *t*‐test for the comparisons between each strain and control strain are indicated as *P ≤ 0.05, **P ≤ 0.01, ***P ≤ 0.001.

In order to summarize the strains development process and their improvements, Fig. 5 compares HT production by our different constructed strains under the best cultivation conditions. The four compared strains are: wild‐type strain BY4743 transformed with empty vectors (control strain), the strain developed in our previous work (Muñiz‐Calvo *et al*., 2020), which overexpressed HpaB + HpaC in episomal plasmid, strain HpaBC (overexpression by integration into the genome; this work) and the best producing strain ARO4*. The absolute HT values were normalized in relation to the HT produced by the control strain (value = 1). Although the simple *E. coli* hydroxylase HpaBC complex overexpression led to markedly increased HT production (around 5000‐fold for the integration of genes), it was the combination of metabolic engineering of the shikimate and Ehrlich pathways together with the hydroxylase complex overexpression that exceeded production by more than 230 000‐fold compared to the control strain.

## Conclusions

Given its antioxidant and beneficial properties, much interest is shown in HT for its use in functional foods, and on pharmaceutical or nutraceutical markets. Unlike chemical synthesis or extraction from natural sources, biotechnological approaches followed to produce lower cost pure HT are very appealing. Unlike our previous work, we metabolically engineered *S. cerevisiae* for high‐level HT production from a simple inexpensive carbon source by integrating the HpaBC complex into the genome and overexpressing several aromatic amino acid pathway‐related genes. Of all the modifications, the single tyrosine insensitive ARO4 allele overexpression (ARO4*) had the strongest effect in the diploid background (BY4743). Nonetheless, the combinatorial overexpression of several ARO genes in the haploid strain (BY4741) enhanced HT production compared to the single ARO4* overexpression. These combinations of overexpressed ARO genes should be transferred as further improvements to the diploid background, which is a higher producer than the haploid version. By further optimization of the medium and culture conditions, the engineered strain increased more than 230 thousand times the HT production of the wild‐type strain with 374.5 mg l^−1^ in shake‐flask experiments in a minimal medium with 160 g l^−1^ of glucose. These results are the highest reported HT titre in *S. cerevisiae* to date. This work lays down the first steps to overproduce HT in yeasts form glucose by a metabolic engineering approach and to further develop a yeast cell factory for HT production. To the aim to increase product yields and to ensure consistent product quality, our next goal will be to study key issues of industrial fermentations and process optimization, which will ensure to scale up these results from the lab bench to the industrial production.

## Experimental procedures

### Strains, media and growth conditions

The yeast strains used in the present work are described in Table S1.

Yeast strains were maintained and grown in YPD medium (20 g l^−1^ glucose, 20 g l^−1^ peptone, 10 g l^−1^ yeast extract) or in SC (1.7 g l^−1^ yeast nitrogen base (YNB) without amino acids and ammonium sulphate (Difco), 5 g l^−1^ ammonium sulphate, 20 g l^−1^ glucose, and an indicated amount of drop‐out powder (Formedium)) supplemented with 16 g l^−1^ agar (Pronadisa) for solid media at 28 °C. The growth medium selected for the HT production experiments was SD (1.7 g l^−1^ yeast nitrogen base, 5 g l^−1^ ammonium sulphate, 20 g l^−1^ glucose). Depending on each strain’s auxotrophic needs (Table S1), media were supplemented with histidine (76 mg l^−1^), methionine (76 mg l^−1^) or leucine (380 mg l^−1^). Strain *E. coli* NZYα (NzyTech) was used as a cloning host for plasmid construction. *E. coli* cells were cultured in LB medium containing 10 g l^−1^ of tryptone, 5 g l^−1^ of yeast extract and 5 g l^−1^ of NaCl supplemented with 100 mg l^−1^ ampicillin to maintain plasmids at 37 °C.

### Plasmid construction

The plasmids and primers herein used are listed in Tables S2 and S3 respectively. For the multicopy integration of the HpaBC complex, genes *hpaB* and *hpaC* and bidirectional promoter TEF1‐PGK1 were PCR‐amplified from plasmids p426GPD‐hpaB, p425GPD‐hpaC and pCfB2628 respectively (Germann *et al*., 2016; Muñiz‐Calvo *et al*., 2020). Primers GV1R‐HpaC, GP1F‐HpaC; PG1R‐TEF1p, PG2R‐PGK1p; GP2F‐HpaB, GV2R‐HpaB were used to carry out these amplifications with Phusion U Hot Start polymerase (Thermo Scientific). In parallel, the vector bearing the Ty1Cons2 sequence from the EasyCloneMulty vector set (Maury *et al*., 2016), pCfB2988, was prepared by sequential treatment with enzymes *Asi*SI (*Sfa*AI) (Thermo Fisher Scientific) and *Bsm*I (New England Biolabs). After purification, the prepared vector and PCR products were mixed and treated with the USER™ enzyme mix (New England Biolabs). After the reaction, the mixture was directly used for bacterial transformation. The successful cloning of different vectors was identified by PCR in *E. coli* colonies using the ADH1_test‐F and CYC1_test‐R primers (Table S3). To verify the exact sequence of the insert, Sanger sequencing (Eurofins genomics) with primers ADH1_test‐F, PGK1p_test‐F, TEF1p test‐F and CYC1_test‐R was performed. Prior to yeast transformation, the resulting integrative vector pCfB2988 HpaBC was linearized by FastDigest *Not*I (Thermo scientific, Vilnius, Lithuania) and the fragment containing the sequences for HpaBC integration was purified from agarose gel.

In order to perform the traditional cloning of ARO genes in 2µ yeast expression vectors, the open reading frames of each gene were PCR‐amplified from the genomic DNA of yeast strain BY4743 using Phusion DNA polymerase (Thermo scientific). Pair of Primers ARO3‐F/ARO3‐R, ARO4‐F/ARO4‐R, ARO7‐F/ARO7‐R and ARO10‐F/ARO10‐R were employed to carry out *ARO3*, *ARO4*, *ARO7* and *ARO10* amplifications respectively. To clone the feedback inhibition‐insensitive ARO genes, the pair of primers ARO3‐F/ARO3K222L‐R, ARO3K222L‐F/ARO3‐R, ARO4‐F/ARO4K229L‐R, ARO4K229L‐F/ARO4‐R; ARO7‐F/ARO7G141S‐R and ARO7G141S‐F/ARO7‐R were used for site‐directed mutagenesis (Landt and Hans‐Peter, 1993). PCR products and vectors were digested with enzymes BamHI and XhoI, gel‐purified and then ligated into the 2µ plasmids of the pRS series (Mumberg and Müller, 1995). The *E. coli*‐positive transformants were selected and plasmids were sequenced with primers GPDPro‐F and CYC1‐R.

About 1–1.5 µg of the linearized fragment from the integrative vector was used for yeast transformation, whereas 200–400 ng of the resulting recombinant 2µ vectors (Table S2) were utilized. Yeast cells were transformed by the PEG/LiAc method according to Gietz and Woods (2002) and selected on selective agar medium according to strain auxotrophic markers and plasmid maintenance needs.

### Cultivations

#### Screening for HT production after HpaBC integration

Twenty‐four single colonies originating from independent transformants were inoculated from solid SC into 800 μl of liquid SC. When the culture was grown to OD ~ 6, 30 µl were transferred to flat‐bottomed 24‐multiwell plates with 1.5 ml of fresh medium, plus 1 mM of tyrosol. Cultures were incubated for 72 h. The final OD_600_ was measured and HT levels were determined by liquid chromatography (HPLC‐PDA).

#### Evaluation of ARO genes overexpression in tyrosol and HT production

Precultures of each strain were grown overnight (o/n) at 28 °C with orbital shaking at 150 rpm in 1.5 ml tubes with 800 µl of SD medium. The next day, 30 µl of the grown culture were inoculated in 1.5 ml of fresh SD medium in 24‐well plates (2 ml capacity). This culture was incubated with constant shaking (300 rpm) at 28 °C for 72 h. The final OD_600_ was measured, and tyrosol and HT levels were determined by HPLC‐PDA.

#### HT and aromatic higher alcohols production during shake‐flask fermentation

In order to evaluate the effect of sampling time and glucose concentration on HT production, the ARO4* strain was inoculated in 1 ml of SD and grown o/n at 28 °C with shaking. The culture was further inoculated in 250 ml flasks containing 50 ml of the same fresh SD medium at two different glucose concentrations (20 or 160 g l^−1^). This culture was incubated with constant shaking at 150 rpm and 28 °C. Samples were taken after 120, 144, 168, 197, 223, 247 and 295 h of growth. The final OD_600_ was measured, and aromatic higher alcohols and HT levels were determined by HPLC‐PDA.

### Determination of aromatic higher alcohols and HT by HPLC‐PDA

For the HPLC‐PDA analysis, samples were diluted 50% v/v with methanol and then centrifuged for 5 min at 7500 *g* at 4 °C. The supernatant was filtered through a 0.22 µm nylon filter before the chromatographic analysis.

Extracellular HT and aromatic higher alcohols (tyrosol, 2‐phenylethanol and tryptophol) were detected by HPLC on an Acquity ARC system core (Waters, Milford, MA, USA) equipped with a photodiode array wavelength detector (Waters 2998 PDA), a quaternary pump, an autosampler and an online degasser. Chromatographic separation was carried out in an Accucore™ C18 (4.6 × 150 mm, 2.6 μm) column (Thermo Fisher Scientific, Waltham, MA, USA) with mobile phases A (0.01% TFA acid in water) and B (acetonitrile). The flow rate was 1 ml min^−1^ and the injection volume was 10 μl. The gradient programme was as follows: 0–18 min, 100% A (0% B), 18–19 min 90% A (10% B), 19–28 min 75% A (25% B), 28–31 min 0% A (100% B) and 31–39 min 100% A (0% B). The column temperature was set at 30 °C and samples were left at 10°C. The PDA detector was set at λ = 210 nm. The identification of all the aromatic higher alcohols and HT was based on their retention times, determined by injecting the reference standards individually and as a mixture. The calibration curves of each analyte, that is, peak area vs. concentration, were linear and data were fitted by the least‐squares method. Linearity was assessed by the least‐squares fitting of the independent six‐point calibration curves. The retention time for HT, tyrosol, 2‐phenylethanol and tryptophol was 9.366 min, 13.112 min, 21.958 min and 22.769 min respectively.

Samples with HT concentration below 200 µg ml^−1^ were analysed with UHPLC‐MS/MS for higher sensitivity as previously described (Muñiz‐Calvo *et al*., 2020). We also confirmed the values obtained in the constructed strains by this accurate method, determining very similar concentrations to the HPLC‐DAD data.

### Statistical analysis

The values are averages of biological triplicates with standard errors. To assess the significance of the differences for each measured compound from groups of strains, a one‐way anova was applied, followed by Tukey’s HSD test (statistical level of significance was set at *P* ≤ 0.05). This analysis was conducted in R (R Development Core Team, 2019). For pairwise comparisons between modified strains in relation to the control strain a Student’s *t*‐test was performed to determine the significance level, with a statistical level of significance set at *P* ≤ 0.05, using GraphPad Prism 7.0 (GraphPad Software, San Diego, CA, USA).

## Conflict of interest

The authors declare no conflict of interests.

## Supporting information


**Fig. S1**. Screening for hydroxytyrosol production at 72 h in SC medium with tyrosol by the different BY4743 strains harbouring HpaBC integrated into several copies. The most productive strain was selected for further studies and is indicated by a triangle.
**Fig. S2**. Effect of knockout *ABZ1*, *TRP2* or *PHA2* on tyrosol production in the BY4743 background. Tyrosol levels produced by the BY4743 wild‐type strain (control), BY4743 mutant for *ABZ1* (Δ*abz1*), BY4743 mutant for *TRP2* (Δ*trp2*) and BY4743 mutant for *PHA2* (Δ*pha2*) were determined after growing in SD medium for 72 h. The tyrosol concentration was determined from the supernatant extracted with methanol and subjected to UHPLC‐MS/MS. Error bars represent the standard deviations calculated from biological triplicates. The values under the same letter are not significantly different according to the Tukey HSD test.
**Fig. S3**. Correlation between tyrosol and 2‐phenylethanol (2‐PE) production by our modified yeast strains.
**Fig. S4**. Hydroxytyrosol production from glucose by strain ARO4* is not explained by the yeast biomass. Strains HpaBC (solid bars) and ARO4*(patterned bars) were cultured in 250 ml flasks with 50 ml of SD with 20 or 160g/L of glucose (grey and pink bars respectively) at 30°C. OD_600_ was measured at different time points. The error bars representing standard deviations were calculated from the biological triplicates of one cultivation. Statistical significance of changes is indicated as ns (not significant, P value > 0.05) or as * (significant, P value ≤ 0.05).
**Fig. S5**. Effect of glucose concentration on aromatic higher alcohols production. Strains BY4743 HpaBC and BY4743 ARO4* (striped and solid bars respectively) were cultured at 30°C in shake flasks filled with SD containing 20 and 160 g/L of glucose (SD‐20 and SD‐160 respectively). The tryptophol (TOL), 2‐phenylethanol (2‐PE) and tyrosol concentration were determined from the supernatant extracted with methanol, and analysed by HPLC‐PDA. The comparisons between strains HpaBC and ARO4* were significantly different in all the time measurements according to the Student’s t‐test (P value ≤ 0.05).
**Table S1**. List of the strains used in this study.
**Table S2**. List of the plasmids used in this study.
**Table S3**. List of the oligonucleotides used in this study.
**Table S4**. Tyrosol, hydroxytyrosol (HT), 2‐phenylethanol (2‐PE) and tryptophol (TOL) production by HpaBC strain transformed with the empty p423GPD vector and the same strain, but overexpressing in the same plasmid one of the following genes: *ARO3*, *ARO4*, *ARO7*, *ARO10*, ARO3*, ARO4* and ARO7* after growing in SD medium for 72 h. Values are represented as mg/L ± SD. Asterisk indicates the overexpression of the mutant variant of the gene (ARO3* ARO4* and ARO7* to indicate ARO3*, ARO4* and ARO7* respectively).
**Table S5**. Tyrosol, hydroxytyrosol (HT), 2‐phenylethanol (2‐PE) and tryptophol (TOL) production by BY4741 HpaBC and the same strain overexpressing several combinations in different 2µ plasmids with the following genes, *ARO3*, *ARO4*, *ARO7*, *ARO10*, ARO3*, ARO4* and ARO7* after growing in SD medium for 72 h. Values are represented as mg/L ± SD. Asterisk indicates the overexpression of the mutant variant of the gene (ARO3* ARO4* and ARO7* to indicate ARO3*, ARO4* and ARO7* respectively). For each compound Tukey HSD test resulted in groups of strains with no significant differences (P value > 0.05) indicated by letters.
**Table S6**. Hydroxytyrosol (HT), tyrosol, 2‐phenylethanol (2‐PE) and tryptophol (TOL) titres produced by the BY4743 control strain (transformed with empty vectors), HpaB + HpaC, HpaBC and ARO4*, after growing in SD with 160 g/L of glucose for 120 h and 240 h. Values are represented as mg/L± SD.
