# Supplementary material for: Metabolic engineering of Saccharomyces cerevisiae for hydroxytyrosol overproduction directly from glucose
Source: Microb Biotechnol. 2021 Oct 24;15(5):1499–510. doi: 10.1111/1751-7915.13957 (PMC9049601; doi:10.1111/1751-7915.13957)
Supplement: Supplementary file 1 — Fig. S1. Screening for hydroxytyrosol production at 72 h in SC medium with tyrosol by the different BY4743 strains harbouring HpaBC integrated into several copies. The most productive strain was selected for further studies and is indicated by a triangle. Fig. S2. Effect of knockout ABZ1, TRP2 or PHA2 on tyrosol production in the BY4743 background. Tyrosol levels produced by the BY4743 wild‐type strain (control), BY4743 mutant for ABZ1 (Δabz1), BY4743 mutant for TRP2 (Δtrp2) and BY4743 mutant for PHA2 (Δpha2) were determined after growing in SD medium for 72 h. The tyrosol concentration was determined from the supernatant extracted with methanol and subjected to UHPLC‐MS/MS. Error bars represent the standard deviations calculated from biological triplicates. The values under the same letter are not significantly different according to the Tukey HSD test. Fig. S3. Correlation between tyrosol and 2‐phenylethanol (2‐PE) production by our modified yeast strains. Fig. S4. Hydroxytyrosol production from glucose by strain ARO4* is not explained by the yeast biomass. Strains HpaBC (solid bars) and ARO4*(patterned bars) were cultured in 250 ml flasks with 50 ml of SD with 20 or 160g/L of glucose (grey and pink bars respectively) at 30°C. OD600 was measured at different time points. The error bars representing standard deviations were calculated from the biological triplicates of one cultivation. Statistical significance of changes is indicated as ns (not significant, P value > 0.05) or as * (significant, P value ≤ 0.05). Fig. S5. Effect of glucose concentration on aromatic higher alcohols production. Strains BY4743 HpaBC and BY4743 ARO4* (striped and solid bars respectively) were cultured at 30°C in shake flasks filled with SD containing 20 and 160 g/L of glucose (SD‐20 and SD‐160 respectively). The tryptophol (TOL), 2‐phenylethanol (2‐PE) and tyrosol concentration were determined from the supernatant extracted with methanol, and analysed by HPLC‐PDA. The comparisons between [file MBT2-15-1499-s001.docx]

**Supplementary Tables**

**Supplementary Table 1**. List of the strains used in this study.

| **Yeast strain** | **Description** | **Source** |
| --- | --- | --- |
| BY4743 | MATa/α *his3∆1/his3∆1* *leu2∆0*/*leu2∆0* *met15∆0*/*MET15* *LYS2*/*lys2∆0* *ura3∆0*/*ura3∆0* | Euroscarf |
| Δtrp2- | BY4743 *trp2::KanMX* | Euroscarf |
| Δabz1- | BY4743 *abz1::KanMX* | Euroscarf |
| Δpha2- | BY4743 *pha2::KanMX* | Euroscarf |
| BY4741 | *MATa his3Δ1 leu2Δ0 met15Δ0 ura3Δ0* | Euroscarf |
| BY4741 Δtrp1- | BY4741 *trp1::KanMX* | This study |
| HpaB+ HpaC | BY4743 p426GPD-hpaB p425GPD-hpaC | Muñiz-Calvo et al., 2020 |
| HpaBC | BY4743 with pCfB2988 HpaBC | This study |
| ARO3 | Strain HpaBC + p423GPD-ARO3 | This study |
| ARO4 | Strain HpaBC + p423GPD-ARO4 | This study |
| ARO7 | Strain HpaBC + p423GPD-ARO7 | This study |
| ARO10 | Strain HpaBC + p423GPD-ARO10 | This study |
| ARO3* | Strain HpaBC + p423GPD-ARO3K222L | This study |
| ARO4* | Strain HpaBC + p423GPD-ARO4K229L | This study |
| ARO7* | Strain HpaBC + p423GPD-ARO7G141S | This study |
| BY4741 HpaBC | BY4741 trp1- with pCfB2988 HpaBC | This study |
| ARO3 ARO4 ARO10 | BY4741 HpaBC + p423GPD-ARO3 + p424GPD-ARO4 + p425GPD-ARO10 | This study |
| ARO3 ARO4*ARO10 | BY4741 HpaBC + p423GPD-ARO3 + p424GPD-ARO4 K229L+ p425GPD-ARO10 | This study |
| ARO3*ARO4 ARO10 | BY4741 HpaBC + p423GPD-ARO3K222L + p424GPD-ARO4 + p425GPD-ARO10 | This study |
| ARO3*ARO4*ARO10 | BY4741 HpaBC + p423GPD-ARO3K222L + p424GPD-ARO4K229L + p425GPD-ARO10 | This study |
| ARO3 ARO4 ARO10 ARO7 | BY4741 HpaBC + p423GPD-ARO3 + p424GPD-ARO4 + p425GPD-ARO10 + p426GPD-ARO7 | This study |
| ARO3 ARO4*ARO10 ARO7 | BY4741 HpaBC + p423GPD-ARO3 + p424GPD-ARO4K229L + p425GPD-ARO10 + p426GPD-ARO7 | This study |
| ARO3*ARO4 ARO10 ARO7 | BY4741 HpaBC + p423GPD-ARO3K222L + p424GPD-ARO4 + p425GPD-ARO10 + p426GPD-ARO7 | This study |
| ARO3*ARO4*ARO10 ARO7 | BY4741 HpaBC + p423GPD-ARO3K222L + p424GPD-ARO4K229L + p425GPD-ARO10 + p426GPD-ARO7 | This study |
| ARO3 ARO4 ARO10 ARO7* | BY4741 HpaBC + p423GPD-ARO3 + p424GPD-ARO4 + p425GPD-ARO10 + p426GPD-ARO7G141S | This study |
| ARO3 ARO4*ARO10 ARO7* | BY4741 HpaBC + p423GPD-ARO3 + p424GPD-ARO4K229L + p425GPD-ARO10 + p426GPD-ARO7G141S | This study |
| ARO3*ARO4 ARO10 ARO7* | BY4741 HpaBC + p423GPD-ARO3K222L + p424GPD-ARO4 + p425GPD-ARO10 + p426GPD-ARO7G141S | This study |
| ARO3*ARO4*ARO10 ARO7* | BY4741 HpaBC + p423GPD-ARO3K222L + p424GPD-ARO4K229L + p425GPD-ARO10 + p426GPD-ARO7G141S | This study |

**Supplementary Table 2**. List of the plasmids used in this study.

| **Plasmid name** | **Description** | **Source** |
| --- | --- | --- |
| p423GPD | Multicopy vector (*HIS3* marker, GPD promoter) | (Mumberg et al., 1995) |
| p424GPD | Multicopy vector (*TRP1* marker, GPD promoter) | (Mumberg et al., 1995) |
| p425GPD | Multicopy vector (*LEU2* marker, GPD promoter) | (Mumberg et al., 1995) |
| p426GPD | Multicopy vector (*URA3* marker, GPD promoter) | (Mumberg et al., 1995) |
| p426GPD-hpaB | p426GPD with the *hpaB* gene from *E. coli,* Amp^R^ | (Muñiz-Calvo et al., 2020) |
| p425GPD-hpaC | p425GPD with the *hpaC* gene from *E. coli,* Amp^R^ | (Muñiz-Calvo et al., 2020) |
| pCfB2988 | Integrative vector for multiple integrations at sites sharing homology with Ty1Cons2 (KlURA3-deg marker) | (Maury et al., 2016) |
| pCfB2988 HpaBC | pCfB2988 with TEF1p::hpaC PGK1p::hpaB | This work |
| pCfB2628 | Bi-directional promoter TEF1p-PGK1p | (Germann et al., 2016) |
| p423GPD-ARO3 | p423GPD with *ARO3* gene from *S. cerevisiae* | This work |
| p423GPD-ARO3K222L | p423GPD with *ARO3^K222L^* gene from *S. cerevisiae* | This work |
| p423GPD-ARO4 | p423GPD with *ARO4* gene from *S. cerevisiae* | This work |
| p423GPD-ARO4K229L | p423GPD with *ARO4^K229L^* gene from *S. cerevisiae* | This work |
| p424GPD-ARO4 | p424GPD with ARO4 gene from *S. cerevisiae* | This work |
| p424GPD-ARO4K229L | p424GPD with *ARO4^K229L^* gene from *S. cerevisiae* | This work |
| p423GPD-ARO7 | p423GPD with *ARO7* gene from *S. cerevisiae* | This work |
| p423GPD-ARO7G141S | p423GPD with *ARO7^G141S^* gene from *S. cerevisiae* | This work |
| p426GPD-ARO7 | p426GPD with *ARO7* gene from *S. cerevisiae* | This work |
| p426GPD-ARO7G141S | p426GPD with *ARO7^G141S^* gene from *S. cerevisiae* | This work |
| p423GPDARO10 | p423GPD with *ARO10* gene from *S. cerevisiae* | This work |
| p425GPDARO10 | p425GPD with *ARO10* gene from *S. cerevisiae* | This work |

**Supplementary Table 3**. List of the oligonucleotides used in this study.

| **Name** | **Sequence (5’-3’)** |
| --- | --- |
| GV1R-HpaC | CGTGCGAUTTAAATCGCAGCTTCCATTTCC |
| GP1F-HpaC | AGTGCAGGUAAAACAATGCAATTAGATGAACAACGC |
| PG1R-TEF1p | ACCTGCACUTTTGTAATTAAAACTTAGATTAGATTGCTAT |
| PG2R-PGK1p | ATGACAGAUTTGTTTTATATTTGTTGTAAAAAGTAGATAATT |
| GP2F-HpaB | ATCTGTCAUAAAACAATGAAACCAGAAGATTTCCG |
| GV2R-HpaB | CACGCGAUTTATTTCAGCAGCTTATCCAGC |
| ADH1_test-F | GAAATTCGCTTATTTAGAAGTGTC |
| PGK1p_test-F | TCTTATCTTGTTTTGCAAGTACC |
| TEF1p test-F | CACACACCATAGCTTCAAAATGTTTCTACTCCTTTTTTA |
| CYC1_test-R | CTCCTTCCTTTTCGGTTAGAG |
| ARO3-F | GTCGTGGGATCCTTATGTTCATTAAAAACGATCACGCCGGTG |
| ARO3K222L-R | GCAGTGACACCTGGCaaTGTGACAGAAAGG |
| ARO3K222L-F | CCTTTCTGTCACAttGCCAGGTGTCACTGC |
| ARO3-R | CCGCTCGAGCTATTTTTTCAAGGCCTTTCTTCTGTTTCTAACACC |
| ARO4-F | GTCGTGGGATCCAAATGAGTGAATCTCCAATGTTCGCTGCCAAC |
| ARO4K229L-R | GCAGCAACACCATGCaaAGTAACACCCATG |
| ARO4K229L-F | CATGGGTGTTACTttGCATGGTGTTGCTGC |
| ARO4-R | CCGCTCGAGCTATTTCTTGTTAACTTCTCTTCTTTGTCTGACAGC |
| ARO7-F | GTCGTGGGATCCATATGGATTTCACAAAACCAGAAACTGTTT |
| ARO7G141S-R | CTAGTGGCAACAGAAgaGAAGTTATTCTTA |
| ARO7G141S-F | TAAGAATAACTTCtcTTCTGTTGCCACTAG |
| ARO7-R | CCGCTCGAGTTACTCTTCCAACCTTCTTAGCAAGTATTCC |
| ARO10-F | AGGTCGTGGGATCCCCATGGCACCTGTTACAATT |
| ARO10-R | TGCGGCCGCTCGAGCTATTTTTTATTTCTTTTAAGTG |
| GPDPro-F | CGGTAGGTATTGATTGTAATTCTG |
| CYC1-R | GCGTGAATGTAAGCGTGAC |

BamHI and XhoI restriction sites for forward and reverse primers, respectively, are underlined. The changes caused by the primers for site-directed mutagenesis are indicated as lower case

**Supplementary Table 4**. Tyrosol, hydroxytyrosol (HT), 2-phenylethanol (2-PE) and tryptophol (TOL) production by HpaBC strain transformed with the empty p423GPD vector and the same strain, but overexpressing in the same plasmid one of the following genes: *ARO3*, *ARO4*, *ARO7*, *ARO10*, *ARO3^K222L^*, *ARO4^K229L^* and *ARO7^G141S^* after growing in SD medium for 72 h. Values are represented as mg/L ± SD. Asterisk indicates the overexpression of the mutant variant of the gene (ARO3* ARO4* and ARO7* to indicate *ARO3^K222L^, ARO4^K229L^* and *ARO7^G141S^*, respectively).

|  | Tyrosol | HT | 2-PE | TOL |
| --- | --- | --- | --- | --- |
| HpaBC | 0.75 ± 0.42 | 0.015 ± 0.005 | 2.49 ± 0.71 | 0.22 ± 0.03 |
| ARO3 | 24.82 ± 0.30 | 0.59 ± 0.27 | 28.26 ± 0.93 | 2.87 ± 0.08 |
| ARO4 | 11.32 ± 0.09 | 0.58 ± 0.05 | 16.34 ± 0.24 | 1.28 ± 0.01 |
| ARO7 | 0.64 ± 0.01 | 0.03 ± 0.00 | 2.54 ± 0.11 | 0.17 ± 0.01 |
| ARO10 | 24.27 ± 0.52 | 0.55 ± 0.08 | 54.13 ± 0.81 | 1.04 ± 0.02 |
| ARO3* | 26.85 ± 0.99 | 1.34 ± 0.15 | 33.08 ± 1.57 | 3.26 ± 0.15 |
| ARO4* | 57.24 ± 2.15 | 2.08 ± 0.27 | 74.91 ± 2.48 | 8.31 ± 0.34 |
| ARO7* | 1.05 ± 0.14 | 0.03 ± 0.003 | 2.80 ± 1.09 | 0.19 ± 0.02 |

**Supplementary Table 5**. Tyrosol, hydroxytyrosol (HT), 2-phenylethanol (2-PE) and tryptophol (TOL) production by BY4741 HpaBC and the same strain overexpressing several combinations in different 2µ plasmids with the following genes, *ARO3*, *ARO4*, *ARO7*, *ARO10*, *ARO3^K222L^*, *ARO4^K229L^* and *ARO7^G141S^* after growing in SD medium for 72 h. Values are represented as mg/L ± SD. Asterisk indicates the overexpression of the mutant variant of the gene (ARO3* ARO4* and ARO7* to indicate *ARO3^K222L^, ARO4^K229L^* and *ARO7^G141S^*, respectively). For each compound Tukey HSD test resulted in groups of strains with no significant differences (P value > 0.05) indicated by letters.

|  | Tyrosol | HT | 2-PE | TOL |
| --- | --- | --- | --- | --- |
| BY4741 HpaBC | 2.27 ± 0.10 g | 0.07 ± 0.00 d | 3.96 ± 0.24 f | 0.99 ± 0.06 f |
| BY4741 ARO4* | 41.61 ± 5.96 ab | 0.73 ± 0.38 bc | 69.96 ± 10.01 abcde | 6.74 ± 0.76 bcd |
| ARO3 ARO4 ARO10 | 26.16 ± 4.98 cdef | 0.59 ± 0.20 cd | 55.64 ± 17.04 bcde | 8.90 ± 1.66 ab |
| ARO3 ARO4*ARO10 | 34.73 ± 2.37 abcde | 0.75 ± 0.20 bc | 69.69 ± 11.31 abcde | 10.10 ± 0.96 ab |
| ARO3*ARO4 ARO10 | 25.46 ± 1.23 def | 0.55 ± 0.07 cd | 52.26 ± 6.22 cde | 8.10 ± 0.47 abc |
| ARO3*ARO4*ARO10 | 36.58 ± 0.57 abcd | 0.82 ± 0.08 bc | 75.66 ± 6.83 abc | 11.02 ± 0.95 a |
| ARO3 ARO4 ARO10 ARO7 | 21.41 ± 2.47 f | 0.52 ± 0.10 cd | 44.37 ± 7.36 e | 6.52 ± 1.58 bcde |
| ARO3 ARO4*ARO10 ARO7 | 37.22 ± 7.25 abc | 1.03 ± 0.27 abc | 72.03 ± 12.12 abcd | 7.87 ± 0.93 abc |
| ARO3*ARO4 ARO10 ARO7 | 24.61 ± 3.19 ef | 0.57 ± 0.13 cd | 47.26 ± 8.88 de | 6.87 ± 2.47 bcd |
| ARO3*ARO4*ARO10 ARO7 | 36.62 ± 5.30 abcd | 1.05 ± 0.26 abc | 71.58 ± 8.39 abcd | 8.41 ± 2.91 ab |
| ARO3 ARO4 ARO10 ARO7* | 26.58 ± 5.43 cdef | 0.72 ± 0.17 bc | 50.06 ± 11.79 cde | 2.69 ± 1.01 ef |
| ARO3 ARO4*ARO10 ARO7* | 45.95 ± 1.91 a | 1.43 ± 0.06 a | 84.48 ± 6.21 a | 4.37 ± 0.64 cdef |
| ARO3*ARO4 ARO10 ARO7* | 33.54 ± 0.78 bcde | 1.20 ± 0.09 ab | 67.30 ± 0.81 abcde | 1.14 ± 0.18 f |
| ARO3*ARO4*ARO10 ARO7* | 43.18 ± 2.88 ab | 1.23 ± 0.12 ab | 80.76 ± 2.43 ab | 3.33 ± 0.27 def |

**Supplementary Table 6**. Hydroxytyrosol (HT), tyrosol, 2-phenylethanol (2PE) and tryptophol (TOL) titers produced by the BY4743 control strain (transformed with empty vectors), HpaB + HpaC, HpaBC and ARO4*, after growing in SD with 160 g/L of glucose for 120 h and 240 h. Values are represented as mg/L± SD.

|  | 120 h | | | | 240 h | | | |
| --- | --- | --- | --- | --- | --- | --- | --- | --- |
|  | HT | Tyrosol | 2PE | TOL | HT | Tyrosol | 2PE | TOL |
| **BY4743** | 0.001 ± 0.001 | 1.677 ± 0.104 | 5.948 ± 0.021 | 0.416 ± 0.002 | 0.002 ± 0.000 | 2.948 ± 0.139 | 8.437 ± 0.124 | 0.388 ± 0.083 |
| **HpaB + HpaC** | 0.473 ± 0.149 | 2.366 ± 0.339 | 6.023 ± 1.833 | 0.468 ± 0.112 | 2.850 ± 0.875 | 4.588 ± 0.915 | 9.219 ± 1.860 | 0.572 ± 0.083 |
| **HpaBC** | 2.402 ± 0.794 | 7.350 ± 1.609 | 5.706 ± 1.300 | 0.533 ± 0.151 | 7.363 ± 2.186 | 12.014 ± 7.131 | 9.140 ± 0.957 | 0.755 ± 0.052 |
| **ARO4*** | 95.119 ± 8.388 | 155.576 ± 3.226 | 308.121 ± 2.609 | 49.387 ± 1.011 | 374.484 ± 52.042 | 210.704 ± 16.677 | 579.669 ± 50.043 | 73.353 ± 5.671 |

**Supplementary Figures**


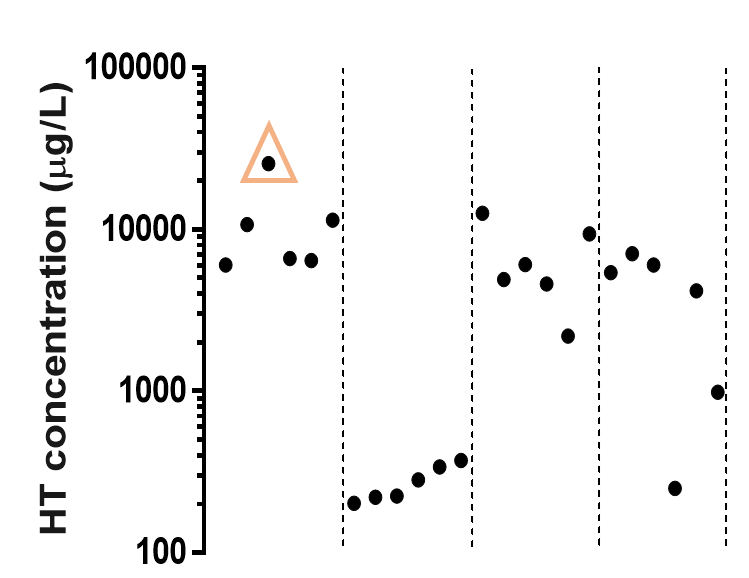


**Supplementary Figure 1.** Screening for hydroxytyrosol production at 72 h in SC medium with tyrosol by the different BY4743 strains harboring HpaBC integrated into several copies. The most productive strain was selected for further studies and is indicated by a triangle.

**Supplementary Figure 2.** Effect of knockout ABZ1, TRP2 or PHA2 on tyrosol production in the BY4743 background. Tyrosol levels produced by the BY4743 wild-type strain (control), BY4743 mutant for ABZ1 (Δabz1), BY4743 mutant for TRP2 (Δtrp2) and BY4743 mutant for PHA2 (Δpha2) were determined after growing in SD medium for 72 h. The tyrosol concentration was determined from the supernatant extracted with methanol and subjected to UHPLC-MS/MS. Error bars represent the standard deviations calculated from biological triplicates. The values under the same letter are not significantly different according to the Tukey HSD test.


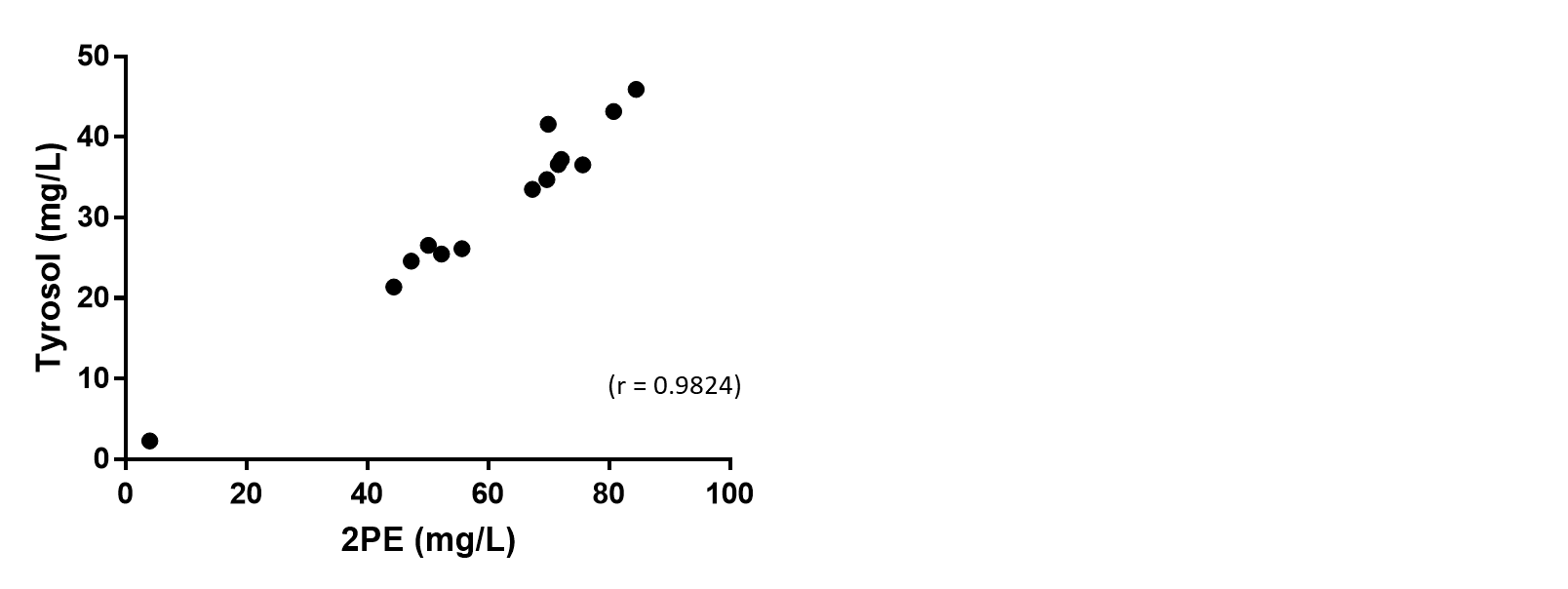


**Supplementary Figure 3.** Correlation between tyrosol and 2-phenylethanol (2-PE) production by our modified yeast strains.

**Supplementary Figure 4.** Hydroxytyrosol production from glucose by strain ARO4* is not explained by the yeast biomass. Strains HpaBC (solid bars) and ARO4*(patterned bars) were cultured in 250 mL flasks with 50 mL of SD with 20 or 160g/L of glucose (gray and pink bars, respectively) at 30 °C. OD_600_ was measured at different time points. The error bars representing standard deviations were calculated from the biological triplicates of one cultivation. Statistical significance of changes is indicated as ns (not significant, P value > 0.05) or as * (significant, P value ≤ 0.05).


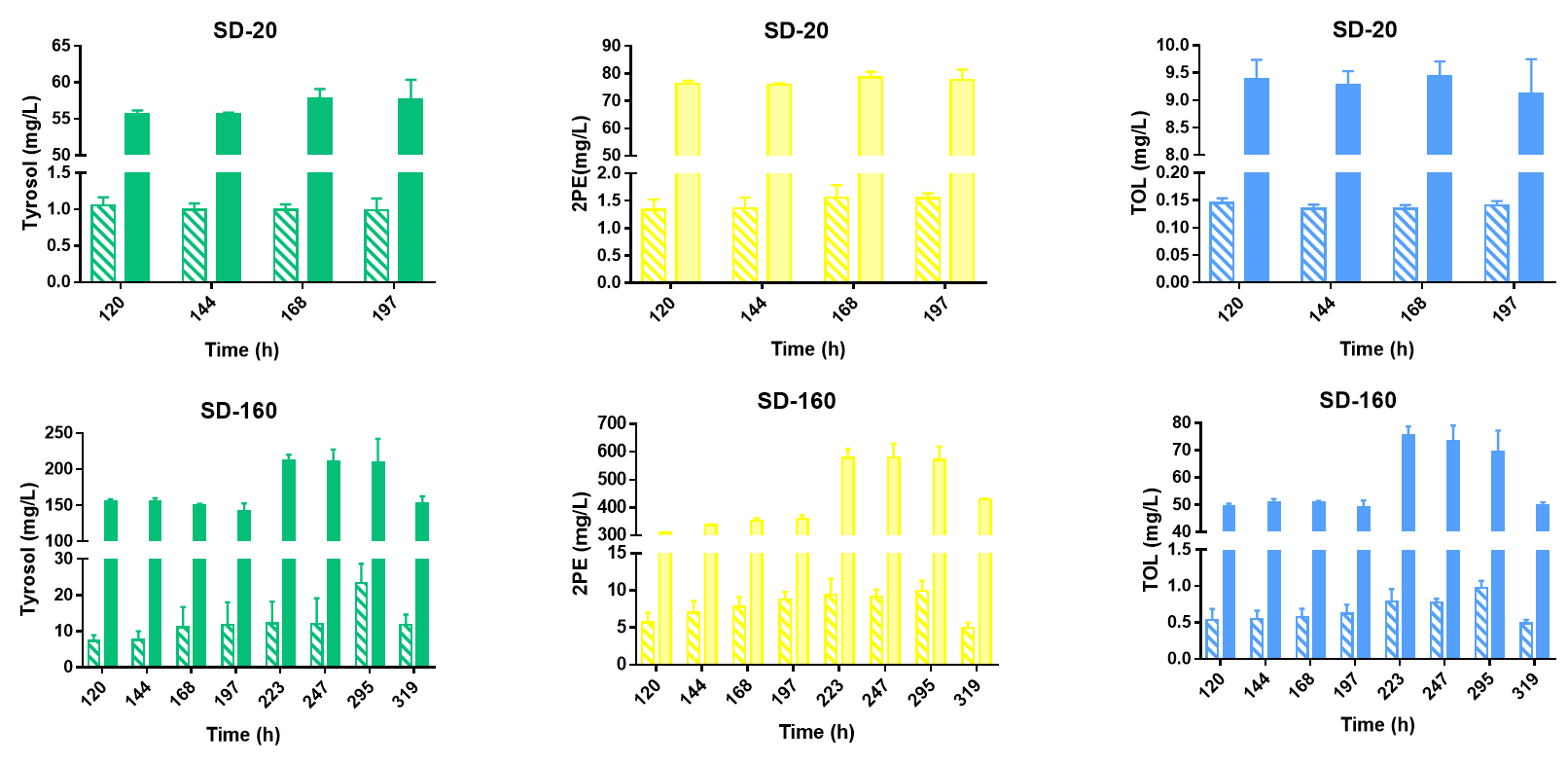


**Supplementary Figure 5.** Effect of glucose concentration on aromatic higher alcohols production. Strains BY4743 HpaBC and BY4743 ARO4* (striped and solid bars, respectively) were cultured at 30 °C in shake flasks filled with SD containing 20 and 160 g/L of glucose (SD-20 and SD-160, respectively). The tryptophol (TOL), 2-phenylethanol (2PE) and tyrosol concentration were determined from the supernatant extracted with methanol, and analyzed by HPLC-PDA. The comparisons between strains HpaBC and ARO4* were significantly different in all the time measurements according to the Student’s t-test (P value ≤ 0.05).
